# Exploring Patient Experiences of Symptom Support From Healthcare Professionals in Acute and Chronic Illness: A Qualitative Study

**DOI:** 10.1111/scs.70210

**Published:** 2026-02-26

**Authors:** Malene Missel, Mary Jarden, Nanna Witting, Helle Pappot, Mai Nanna Schønau, Maria Camilla Mathiasen, Camilla Corvinius, Pernille Orloff Donsel, Melissa Culligan, Giulia Locatelli, Karin Piil, Linda Kahr Andersen

**Affiliations:** ^1^ Department of Cardiothoracic Surgery Copenhagen University Hospital—Rigshospitalet Copenhagen Denmark; ^2^ Department of Clinical Medicine University of Copenhagen Copenhagen Denmark; ^3^ Department of Haematology Copenhagen University Hospital Rigshospitalet Copenhagen Denmark; ^4^ Department of Neurology, Copenhagen Neuromuscular Center Copenhagen University Hospital—Rigshospitalet Copenhagen Denmark; ^5^ Department of Oncology Copenhagen University Hospital—Rigshospitalet Copenhagen Denmark; ^6^ Thoracic Surgery Research, Department of Thoracic Medicine and Surgery Temple University Hospital Philadelphia Philadelphia Pennsylvania USA; ^7^ Department of Medicine and Surgery University of Milano‐Bicocca Monza Italy

**Keywords:** acute and chronic illness, healthcare professionals, patient involvement, patient‐centred care, phenomenology, qualitative study, Ricoeur, symptom experiences, symptom science

## Abstract

**Background:**

Symptoms are a central part of living with illness, and healthcare professionals play a crucial role in supporting patients in the managing of these. Symptom support is not only about alleviating physical discomfort but also about addressing the subjective and existential dimensions of illness. Understanding how patients experience such support is essential for strengthening communication, dignity, and person‐centred care.

**Aims:**

This study aims to explore the meaning of patients' experience of support from HCP in managing symptoms, and how this meaning shapes their understanding of symptoms and subsequent strategies.

**Methods:**

A qualitative design was applied. In‐depth interviews were conducted with 10 patients diagnosed with acute or chronic illness and receiving care in hospital and outpatient settings. Data were analysed using a phenomenological hermeneutic approach inspired by Ricoeur.

**Findings:**

Three themes were identified. *Caught in the gaps of continuity in care* describes how patients experienced symptom support as fragmented and inconsistent, shaped both by organisational structures and the professionals they encountered. *Dialogue, relationship and professional knowledge as a vulnerable space* highlights how encounters with healthcare professionals were perceived as fragile moments, where recognition, time, and competence could either foster trust or leave patients feeling exposed. *Between silence and support in the healthcare encounter* reflects how organisational structures and clinical routines often failed to address patients' concerns, creating silent spaces where symptoms and existential needs were overlooked.

**Conclusions:**

Patient experiences reveal how symptom support is shaped by both relational vulnerability and systemic fragmentation. When symptoms are overlooked, patients feel abandoned and burdened with coordinating their own care. Strengthening relational continuity, clarifying roles across professions explicitly to patients, and prioritising dialogue and empathy in clinical encounters are crucial steps toward ensuring person‐centred symptom management that promotes dignity and well‐being.

## 1 Background

Every clinical meeting begins with a patient's first expression—a moment that carries both clinical significance and human meaning. This initial utterance should not only be heard but also responded to, like an outstretched arm reaching toward the healthcare professional (HCP). Such encounters unfold in a relational space, where mutual understanding, recognition, and care unfold. In this space, care extends beyond addressing the patient's physical needs; it also involves acknowledging their subjective experience and the existential dimensions of their symptoms [1, 2, 3, 4].

In contemporary healthcare, understanding patients' subjective experiences of symptoms is crucial for effective care. As medical advancements prolong survival, the complexity of managing both acute and chronic illness has increased [5, 6]. Symptoms are not merely physiological signs of a disease—they are embedded in patients' lived experiences, significantly influencing their identity, autonomy, and daily functioning [7]. Symptoms like fatigue, pain, and breathlessness carry existential weight, often challenging a patient's sense of control and reshaping their relationship with their body and surroundings [8, 9, 10, 11, 12, 13, 14].

Research emphasises the critical role of HCP in acknowledging both the physical and existential dimensions of symptoms [7]. In line with Carel's concept of epistemic humility [15, 16, 17], effective care requires HCP to remain open to understanding the patient's subjective experience, while also integrating their professional knowledge. Creating reflective spaces where patients feel heard and validated can help foster person‐centred care that addresses not only the medical but also the emotional and existential challenges posed by the illness. Such support can help patients regain a sense of control, resilience, and dignity in their lives [18, 19]. Previous studies have demonstrated that patients often report unmet needs related to symptom communication, continuity of care, and psychosocial support, particularly in transitions between treatment phases and care settings. Qualitative studies have highlighted challenges related to feeling unheard, lack of continuity, and uncertainty about when and how to raise symptom concerns, while quantitative research has primarily focused on symptom burden, satisfaction, and predefined outcome measures. Despite this growing recognition in research, theory and policy, there remains limited empirical, person‐centred knowledge of how patients actually perceive and experience the support provided by HCP in managing their symptoms in everyday clinical practice. While previous studies have explored patient satisfaction and clinical outcomes [20, 21, 22, 23, 24, 25], little is known about how clinical interactions influence patients' understanding and communication of their symptoms and their coping strategies. Such knowledge is essential for developing ethically grounded, clinically relevant and contextually sensitive approaches to symptom management that are informed by patients' lived experiences rather than solely by professional or biomedical perspectives [17, 26, 27].

This study is part of The Precision Symptom Care Research Program (PROSPER), a multi‐disciplinary initiative at Copenhagen University Hospital, Rigshospitalet, Denmark, that aims to understand and improve symptom management across illnesses and care contexts. By exploring how patients perceive and experience the support provided by HCP in managing symptoms, and how they interpret the meaning of such support in relation to their everyday lives, this study seeks to generate insights into how communication may be enhanced, how empathetic and person‐centred interventions can be informed, and how dignity and well‐being may be promoted in clinical practice.

## 2 Aim

This study aims to explore the meaning of patients' experience of support from HCP in managing symptoms, and how this meaning shapes their understanding of symptoms and subsequent strategies.

## 3 Method

The study is grounded in a phenomenological hermeneutical approach, drawing on Ricoeur's philosophy of interpretation [28, 29, 30].

### 3.1 Participants and Recruitment

Ten patients were purposively selected [31] to represent diverse perspectives on symptom experiences. The study was conducted in Denmark, and participants were recruited from specialised outpatient clinics at Danish university hospitals. In line with qualitative sampling principles, variation was sought in relation to diagnosis, treatment context, and illness trajectories in order to capture a breadth of experiences across different clinical settings. Inclusion was based on participants having lived experience of symptom management in the context of serious illness (acute and/or chronic), being able to reflect on and articulate their experiences, and being willing to participate in an in‐depth interview. The sample included patients with lung cancer (thoracic surgery), gynaecological or brain cancer (oncology), and myasthenia gravis (neuromuscular clinic). Time since diagnosis ranged from 2 weeks to 26 years across participants. Recruitment, facilitated by four authors (KP, LKA, MCM, MM), occurred during scheduled visits or via prior HCP connections. All invited patients agreed to participate. Interviews were conducted between March and July 2024, and participant characteristics are shown in Table 1.

**TABLE 1:** Participant overview.

Participant	Gender	Age	Living situation	Education	Employment status	Diagnosis	Time since diagnosis
Patient 1	Male	69	Lives with partner	Lower secondary school	Early retirement due to illness	Myasthenia gravis	3 years
Patient 2	Female	58	Lives with partner	Primary school	On sick leave	Myasthenia gravis	11 months
Patient 3	Female	78	Lives alone	Dietician	Retired	Myasthenia gravis	26 years
Patient 4	Female	62	Lives with partner	Office clerk training	Early retirement due to illness	Ovarian cancer	8 years
Patient 5	Female	41	Lives with father	Primary school	Early retirement due to illness	Ovarian cancer	6 years
Patient 6	Female	45	Lives alone	Self‐employed	On sick leave	Brain cancer	3,5 years
Patient 7	Female	67	Lives with partner	Trained kitchen assistant	Retired	Lung cancer	2 weeks
Patient 8	Female	53	Lives with partner and children	Researcher	Early retirement due to illness	Lung cancer	10 years
Patient 9	Male	68	Lives with partner	Primary school teacher	Retired	Lung cancer	1 years
Patient 10	Female	63	Lives with partner	Vocational education	Early retirement due to illness	Lung cancer	2 years

### 3.2 Data Collection

Drawing on Ricoeur's view that we first encounter the world pre‐reflectively [32], we employed a narrative approach in individual interviews to illuminate patients' lived experiences [28, 33]. Patients were encouraged to narrate their experiences of illness, symptoms, and support in their everyday lives. Open‐ended questions served as entry points to elicit lived experiences, while follow‐up prompts were used to deepen reflection on meaning, bodily experience, relational dynamics, and the impact on daily life (see Table 2). Interviews were dialogical, unstructured, and shaped by patients' own narratives. They were conducted face‐to‐face by five experienced qualitative researchers and clinicians (MCM, LKA, POD, CC, MM), all trained in qualitative interviewing. The interviewers had professional backgrounds in nursing and physiotherapy and extensive experience with patient communication. To enhance consistency across interviews, the interviewers discussed the interview approach and guide prior to data collection, and analytic reflections across interviews were continuously discussed within the research team. Interviews lasted 30–90 min (median 60 min) and were audio‐recorded and transcribed verbatim. The length of interviews varied according to patients' narratives and engagement, and depth was supported through follow‐up questions and reflective probing rather than fixed duration. Ethical considerations, including informed consent, were addressed, and data were pseudonymized to ensure confidentiality [34].

**TABLE 2:** Interview questions.

Can you tell me about what it is like for you to live with your illness?
Can you tell me how you experience symptoms?
Can you tell me how you manage the symptoms you experience?
Can you tell me about when you first noticed these symptoms and how they have changed over time?
Can you tell me how you talk with healthcare professionals about the symptoms you experience?
Do you experience symptoms that are not addressed by healthcare professionals? Can you tell me about them?
Can you tell me about the support you've received from healthcare professionals regarding your symptoms?

### 3.3 Data Analysis

Following Ricoeur, human expressions hold ambiguous traces rather than fixed meanings; interpretation aims to disclose a ‘world’ of human possibilities [28, 35, 36, 37]. In Missel & Birkelund [28], the approach is described as an interpretive orientation informed by Ricoeur rather than a stepwise procedural method; the present study operationalizes this orientation through interpretive levels described below.

Patient narratives were interpreted in three iterative stages. First, MM and LKA engaged in repeated readings to gain an overarching, ‘naive’, understanding of the text as a whole, which functioned as an orienting interpretive stance guiding subsequent analyses. As this step is preliminary and continually revised during analysis, it is not presented as a separate set of findings. Second, a structural analysis examined the text's internal composition (distanciation), breaking it into meaningful units and identifying patterns and themes beyond literal meaning. The Findings section presents this structural analysis in thematic form. This process involved ongoing dialogue between MM and LKA, and analytic reflections were documented throughout. Finally, a comprehensive interpretation integrated the naïve understanding and structural analysis with our pre‐understandings, relevant theory, and existing literature, allowing for a critical and reflective interpretation of the narratives as a whole and restoring the text to dynamic communication. The Discussion section develops this comprehensive interpretation by bringing findings into dialogue with theory.

Preunderstanding was addressed through reflexive team discussions across disciplinary perspectives (nursing, physiotherapy, medicine, oncology, surgery, and neurology), challenging assumptions and strengthening interpretive depth. All authors contributed to and approved the interpretation, ensuring both theoretical grounding and clinical relevance. This interpretive process sought to illuminate universal insights into the lifeworld dimensions of symptom support [29, 36].

## 4 Findings

Three themes presented in this section express how the narratives illuminate experiences of symptom support in encounters with HCP.

### 4.1 Caught in the Gaps of Continuity in Care

A central theme across the interviews highlights how experiences of symptom support are perceived as fragmented and inconsistent, shaped by which HCP patients encounter, the department they are affiliated with, and whether enough time is taken to listen to them. This fragmentation is particularly evident after acute treatment, where the narratives convey a clear sense of support during surgery or chemotherapy, but a lack of follow‐up and a feeling of being left to navigate the system alone. For patients with chronic illness, such as myasthenia gravis, who maintain ongoing contact with specialised clinics, the picture is not necessarily more cohesive. Here, too, the narratives reveal encounters with different HCP and a lack of continuity. The narratives illuminate a desire for more specialised support in daily life, for example access to a physiotherapist with disease‐specific knowledge. At the same time, this wish was closely tied to the importance of the physical encounter itself. As one account illustrates: ‘*I would have liked a physiotherapist who specialises in this. There must be others who've had the same problem. In the United States, you can get exercises and guidance, but here you're on your own … I would have liked a physical person to see me, because I wasn't 100% sure I was doing it right. It's different talking to a person than to a computer. It helps, but I miss someone who can see me and guide me properly*’. This shows that patients' needs are not only about access to disease‐specific expertise, but also about the reassurance and guidance that emerge in face‐to‐face encounters.

Across diagnoses, the narratives reveal a need to take responsibility for communicating symptoms, ensuring follow‐up and initiating contact. This role as ‘*the coordinator*’ can be burdensome during a vulnerable phase marked by fatigue, side effects, and existential strain. One account illustrates this: ‘*I have to be the coordinator myself … it would be nice if I could just be the patient, and the doctor would tell me what to do*’. This exhaustion, compounded by uncertainty about whether symptoms are ‘important enough’ to discuss, can lead patients to minimise or even neglect their symptoms.

The narratives convey a strong desire for a consistent point of contact, someone who knows the illness journey and can offer support across its various phases. The lack of continuity can decrease patients' motivation to engage in their own treatment and well‐being, as expressed in this account: ‘*It's someone new every time, and that's a problem. It makes me less engaged. I'm sorry to say it, but I care less about the treatment I get here. I do it out of obligation, because I promised*’. Especially after cancer treatments, feelings of being ‘dropped’ or abandoned by the healthcare system are reinforced as shared here: ‘*You're just dropped, and then you're on your own*’, reflecting a common sentiment that the system's fragmentation leaves patients feeling abandoned and disconnected. This lack of continuity creates not only practical challenges but also doubt and uncertainty. The sense of a fragmented system is further amplified by responsibilities being divided among different HCP, and the broader context of the patient's life and illness experience being overlooked: ‘*It was mostly the nurses who talked to me about how I was doing at home. The doctors' conversations were more about protocols and treatments. It was all very divided*’. Such fragmentation can make it unclear whom to approach with concerns that fall outside a narrow treatment framework.

Care is experienced as fragmented across the narratives, with movement between departments and sectors lacking coherence, reinforcing a sense of being left alone to create continuity and oversight. This is particularly evident during transitions—between hospital and community care, between surgery and follow‐up, or when symptoms do not fit into a clinical category. The narratives call not only for better communication between sectors but also for a greater willingness from HCP to consider the overall picture and engage with the patient's broader life situation: ‘*It's like the illness is the most important thing, and everything around it … that's for the community or your GP, if you have one. But think about how much money they spend to make people cancer‐free, and then they don't follow through. There's a silo mentality. The surgeon can operate but doesn't deal with anything else. Then you go somewhere else, and they say, “We can't do more”. Things fall between the cracks because communication and coordination aren't good enough*’.

The interviews also showed that when continuity does succeed, it makes a significant difference. One account illuminates having a consistent doctor: ‘*I have a doctor at X Hospital. He takes his time. He asks if there's anything I'm worried about. There's empathy. He's engaged in what's good for me. He's really good at keeping track of where I'm at. I'm really happy with him*’. Such experiences are characterised by trust, mutual understanding, and a sense of continuity over time, where patients feel acknowledged within the broader trajectory of their illness rather than reduced to isolated clinical encounters.

### 4.2 Dialogue, Relationship and Professional Knowledge as a Vulnerable Space

A key theme that emerged in the interviews is the crucial role that dialogue and the relationship with HCP play in patients' experiences of symptom support. Feeling seen, heard, and taken seriously is vital for patients to feel supported in managing their symptoms. When professional expertise is combined with empathy and the ability to create a safe space, the narratives reveal how patients perceive these conversations as meaningful. They feel recognised as individuals with unique needs within the clinical encounter, through dialogue that acknowledges their concerns, uncertainties, and lived experience. One narrative account shared: ‘*You are the doctor. You are the one who knows. You are the one with the experience. You are the one who has to be there and tell me what can be done*’. This reflects a desire of the patients for the HCP to take responsibility for the conversation, especially in vulnerable situations where uncertainty is high.

The need for support is particularly high in the early phase after diagnosis, when uncertainty is at its peak. The analysis emphasised how communication during this initial period shapes both patients' trust in the system and their own ability to navigate it. When serious information is delivered without preparation or face‐to‐face contact, it can feel harsh and increase feelings of vulnerability: ‘*It was a bit rough to be told over the phone that I had a 2.5 cm tumor without it ever having been discussed. You're vulnerable then, because you're afraid they'll tell you it's something you'll die from*’. The narratives also illuminate how patients' ability to ask questions and process information often emerges later, once the initial shock has subsided. This points to the need for differentiated support across different phases of care, and for HCP to take greater responsibility in the early, more vulnerable stages and delivering information with sensitivity and acknowledging that patients' cognitive and emotional capacity may be limited during these times.

Illuminated in the narratives are also expressions on how consultations can feel too technical, rushed, or lacking in emotional awareness: ‘*It would be nice if they told you how to help yourself—then you'd also be more motivated. But sometimes it feels like they only see the disease, not the person behind it. I told the doctor what I had eaten, but no one says how it relates to the medication. It puzzles me, and it actually makes me nervous*’. Such encounters create distance and insecurity, leading patients to hold back questions for fear of appearing ignorant or bothersome: ‘*You don't always understand what they say, and you don't want to seem stupid*’. Additionally, when patients face new HCP in each encounter, it becomes even harder to establish the relational safety needed to share personal or sensitive symptoms: ‘*The main problem is that I don't have the same doctor every time, so it feels like starting over again and again. And then the symptoms that are more private than just saying I have neck pain—they're harder to bring up if it's not someone you know*’. This highlights the need for HCP to be proactive in establishing a trusting dialogical space, both by sharing professional knowledge and by actively opening conversations that invite patients' perspectives. In the analysis it is revealed how such proactive support can look in practice: ‘*It was me who reached out about the symptoms that appeared after the treatment, but I felt they always took my concerns seriously and talked with me about it*’.

Finally, the narratives illuminate how symptoms are rarely just physical, as they are closely connected to existential reactions. Even a minor symptom like coughing can trigger worry about disease progression, leading patients to overthink and search for answers independently: ‘*When I feel something physically, it immediately goes to my head. I can tell I'm overthinking. And then I Google it…*’. The analysis reveals patients' desire for HCP to take an interest in how symptoms impact their daily lives, identity, and mental state—not just their biological significance. When this existential dimension is overlooked, it creates a disconnect between the patient's personal experience and the clinical framework, leaving them to assess how serious a symptom is and whether it's ‘important enough’ to seek help. In the narratives, patients describe experiences of trying to fit into a predefined understanding of illness and symptoms: ‘*It's like they have a checklist of what's right, and the rest doesn't fit. You have to fit into the box*’. This illustrates a sense that support is not always based on the patient's specific experience, but rather on standardised expectations of what symptoms ‘should’ be like.

### 4.3 Between Silence and Support in the Healthcare Encounter

A recurring theme in the interviews is the tension between silence and support in how communication about symptoms is experienced by patients. One account shared: ‘*During the treatment itself, I felt I got good help. But afterwards—when it comes to late effects, energy management, and how to move forward—you're on your own. It's like the medical and the human worlds are separate, and the latter is something you have to figure out yourself*’. This highlights the divide between the medical and human aspects of care, which can feel disconnected and leave patients to manage the broader implications of their illness alone.

The narratives also illuminate how symptoms such as fatigue, pain, sleep disturbances, and emotional distress play a significant role in patients' daily lives, yet these concerns are often not addressed in conversations with HCP: ‘*For the first time, I had written down a list of points to ask my neurologist about, but there wasn't time. She was kind, but stressed, and it became her agenda instead of my questions. I just wanted to be heard. It's frustrating when you lie awake at night and don't even get a chance to mention what's on your mind—I just want to be heard … it makes you feel sad and frustrated*’. When conversations are shaped by professional agendas and limited time, the analysis shows how patients feel their perspectives are not invited, leaving their symptoms and experiences unspoken: ‘*You (HCP) can't walk in as a professional and in 10 s think you know everything. You don't. They need to be interested in me, not just in the disease. That's the only way they can help with both treatment and the illness*’. This sense of ‘tacit silence’ is intensified when consultations are brief or conducted over the phone. Silence, in this case, becomes more than the absence of words—it reflects a lack of relational and temporal space to discuss what patients feel is important: ‘*You get a letter saying, ‘Everything looks fine. You will be scheduled for another scan’. But no one asks how you're doing’. ‘The letter says the conversation will last 10 min. So, what are you supposed to say?*’

Not all patients shared this experience. Other accounts conveyed experiences of being consistently met with interest and support for their symptoms: ‘*If I have something, we talk about it, and they suggest something. I get lotions, aids, blood tests—I always get some help, I can see that. They always ask how I'm doing*’. The narratives also emphasise how moments of human connection and care made a lasting impact: ‘*Right after the surgery, I couldn't even look at my scar—I felt disgusting and different. But the nurses were completely professional and didn't make me feel wrong. I was really grateful for that*’. These encounters created a sense of trust and safety, allowing patients to feel free to share their symptoms and receive concrete support. The analysis highlighted how longer consultations, face‐to‐face meetings, and familiar HCP helped patients create meaningful and supportive conversations: ‘*Those consultations where I came in and we could talk about everything—those were good. But that was a long time ago*’. These experiences underscore that the presence of dialogue and relational connection makes a difference, and that its absence can leave patients feeling alone. It is not just knowledge or treatment that patients seek, but the opportunity to be heard and supported in what feels difficult.

The interviews also reveal patients' resourcefulness and ability to develop problem‐solving strategies. Patients developed their own ways of managing symptoms, through physical activity, rest, mental techniques, or support from family and friends. While patients tried to help themselves, they emphasise the importance of professional support: ‘*I've really tried to help myself—and of course, you want to. But if you get support and strength, you just get through it faster. The professionals have experience, they could help you hold it all together. It's not just a medical disease, it's also a big task to get back to life afterwards*’. Other accounts also mentioned the importance of peer support, both online and in hospital settings: ‘*There are so many of us here at the hospital with cancer, and we talk about what we've been through and what we do. I share my experiences, and it often helps, especially for those who are newly diagnosed*’. Peer exchange may offer not only practical advice but also a sense of belonging and understanding, helping to compensate for the silence and fragmentation patients sometimes experience in the healthcare system.

## 5 Discussion

Our findings portray a healthcare system organised around brief encounters and narrow frameworks, where continuity often seems to depend on chance. This creates an experience of being left to fend for oneself, with support that is not proactive but contingent upon the patient's own resources and ability to insist. This issue does not seem entirely new. Research over the past several years has highlighted patients' experiences of lacking continuity, presence, and dialogue throughout their treatment [38, 39, 40, 41]. However, as the findings of this study suggest, the issue remains unresolved, likely because it is inherently complex and not readily addressed through simple solutions.

Patients' experiences of support are shaped both by systemic conditions and by the individual professional they meet. Organisational frameworks, time constraints, and professional background influence whether a consultation becomes meaningful or a missed opportunity, as illustrated in our findings where patients described rushed consultations and uncertainty about who was responsible for addressing their concerns. For instance, a rehabilitation nurse may focus on late effects, while a physician emphasises acute treatment, and limited time can leave concerns unaddressed. Prior research confirms how structural and relational factors—such as continuity, time pressure, and specialisation—affect communication and attention to symptoms [42, 43]. These findings underscore that symptom support is fragile and uneven, shaped not only by systemic fragmentation but also by variability in professional focus and expertise across encounters.

Over the past decades, policymakers, HCP, and patient advocacy organisations have worked actively to promote patient involvement and shift the healthcare system toward partnerships between patients and professionals. Slogans such as ‘patient‐centred care’, ‘patients first,’ and ‘the patient as partner’ have shaped both political visions and professional ideals [44, 45, 46]. In many Western countries, this development stems from the recognition that a paternalistic approach to health and treatment is both ethically problematic and clinically insufficient and that the patient's perspective is central to making relevant decisions during illness [45]. Although long discussed, patient involvement has only recently gained a stronger foothold in practice, supported by works such as *Whole Person Care: A New Paradigm for the 21st Century* [47] and Coulter's often‐cited phrase *No decision about me, without me* [48].

Many clinical environments have sought to operationalise patient involvement through tools such as patient‐reported outcomes (PROs) and the Patient Concerns Inventory (PCI) [49, 50]. While these initiatives can make patients' needs more visible, the findings of our study show that structural and cultural barriers remain. Collecting PROs or asking questions is insufficient without time, continuity, and genuine openness. Literature on patient‐centred communication highlights dialogue, respect, and individualised support as essential [38, 39, 40]. Yet, in practice, involvement often appears as a formal concept rather than lived reality. As Entwistle et al. [39] argue, patient involvement should be seen not just as a technical practice but as an ethical relationship grounded in recognition, responsiveness, and trust—elements also emphasised by recent studies [18]. The presented findings also suggest that patients often expect the physician to ‘take care of everything’, while the roles of other professionals remain unclear. This highlights a communicative and organisational challenge; without transparency about responsibilities, patients may feel insecure, overburdened, or hold unrealistic expectations [51, 52].

These challenges highlight the need to rethink what patient involvement truly requires in clinical practice. In this context, Mol's concept of *the logic of care* [53] provides an expanded understanding of patient involvement. Mol criticises the idea that involvement primarily means giving patients more choices—a *logic of choice*, where the ideal patient is informed and actively making decisions. In contrast, the logic of care emphasises practical ethics that arise from the concrete collaboration between patient and HCP. Here, it is not about ideals, but about situation‐specific adjustments, negotiations, and adaptations, where professional knowledge and the patient's life situation are brought into play and aligned. It is a continuous process, where both needs and solutions evolve in the interaction between people, rather than in standardised protocols. This approach aligns with the findings in our study, where patients specifically call for continuity, responsiveness, and relational support rather than more choices or decision points. It is not the availability of choices in itself that creates security, but the ongoing adjustment and presence that allows for understanding and managing symptoms in context and over time—which is precisely the core of Mol's concept of care—as also seen in recent work on healthcare preferences and values [54, 55, 56].

Toombs [57, 58] and Carel [15, 17], among others, have shown how illness fundamentally alters a person's bodily experience and perception of the world, an aspect often overlooked in systems designed to identify and act upon measurable deviations. For Toombs [57, 58], illness is not just a physical interruption, but an existential disruption that disturbs the self and separates the patient's lifeworld from that of the HCP. This asymmetrical relationship becomes central in the findings of this study, where patients describe the experience of not being met, heard, or understood—or having their symptoms reduced to biological phenomena without existential significance. Carel [16, 17] further elaborates that illness also contains an epistemic dimension; the patient risks experiencing what she calls epistemic injustice—that is, being deprived of authority over their own experience. When symptoms are not recognised, or when no one inquiries about them, patients' knowledge and experience are invalidated. This is clearly reflected in our findings, where patients describe withholding symptoms because they are unsure if they are ‘important enough’ or lack the energy to explain them. The result is not only uncertainty but also loneliness and the feeling of having to manage their own care. It is crucial, therefore, for HCP to recognise symptoms not just as objective signs but also as subjective experiences that must be understood in context. This reflects not only a lack of clinical attention but also a structural silence regarding what is most important for the patient [16, 59, 60].

This form of existential and epistemic vulnerability calls for a different type of response, one that is not solely based on evidence and treatment algorithms. It calls for what Toombs describes as the moral obligation to listen [57, 61]—not merely hearing about symptoms but listening to how they are experienced and understood in the patient's everyday life. It is in this listening that dialogue, relationship, and professional expertise can come together to create the support that patients in our study are seeking. However, as the findings show, this requires time, continuity, and the willingness to understand illness as more than a biological deviation—as a disruption in life, identity, and the relationship with the world.

At the same time, the challenges patients talk about are not only relational but also structural and clinical. Some of the uncertainty and lack of support experienced by patients in our study may stem from gaps in clinical knowledge. For example, HCP often lack clear tools or solutions for managing fatigue effectively [62, 63, 64, 65]. Moreover, healthcare systems oriented toward efficiency and reduced waiting times may leave little space for the presence and dialogue that patients seek. This highlights a broader tension: how can highly specialised professionals also attend to patients' existential and psychosocial concerns? These questions call for a shared responsibility, where symptom support is recognised as both a clinical and relational task—demanding responsiveness at both the systemic and individual levels.

### 5.1 Strengths and Limitations

In phenomenological health research, validity, trustworthiness, and transferability are complex concepts [66, 67]. Our study aimed to explore shared meanings behind patients' symptom experiences, focusing on universal features of human existence in their interactions with HCP. Validity was pursued by interpreting patients' descriptions through Ricoeur's theory of narrative and interpretation [28, 29, 36], identifying structures and patterns while acknowledging individual variation [68, 69]. Trustworthiness was supported by rigorous data collection and analysis, as well as the interdisciplinary background of the research team, which enriched interpretations and enabled reflexive dialogue across perspectives. At the same time, we acknowledge that interpretation is inevitably shaped by the researchers' professional and theoretical positions, which may have influenced the analytic focus. The small sample allowed for in‐depth exploration; however, the findings may primarily reflect the perspectives of participants who felt comfortable sharing and reflecting on their experiences in an interview setting [66]. Furthermore, participants were recruited from specialised outpatient clinics at university hospitals, which may shape experiences of symptom support and limit transferability to other healthcare contexts.

Transferability in phenomenology lies in offering detailed accounts that may resonate with others in similar contexts [66]. Our diverse sample across diagnoses and treatments provided a broad view of symptom experiences, though variation in conditions may have shaped individual accounts. We emphasised universal aspects of symptoms rather than illness‐specific details, which may also mean that certain contextual nuances are less visible in the analysis. Limitations include uneven gender distribution (two men) and limited age variation, which should be considered when interpreting the findings. The involvement of multiple interviewers may have influenced the interview dynamics; however, this was addressed through shared training, ongoing dialogue within the research team, and joint analytic discussions, which supported reflexivity and trustworthiness. Differences in time since diagnosis may have influenced participants' ways of describing and reflecting on symptoms, as experience with illness can shape how symptoms are interpreted and communicated.

## 6 Conclusion

This study highlights a persistent gap between knowing what matters to patients and translating this knowledge into everyday clinical practice. Patients' experiences reveal a fundamental existential need for recognition, empathy, and clear communication in encounters with HCP. The findings suggest that symptom support depends not only on clinical knowledge but also on the communicative competencies of HCP and their ability to attend to patients' lived experiences beyond biomedical concerns. A predominant focus on biomedical aspects of care may contribute to patients' existential and psychosocial concerns receiving less attention. Symptom support is further shaped by organisational conditions such as continuity, time, and clarity of professional responsibilities. Strengthening relational continuity, supporting communicative competencies, and creating organisational space for addressing more than physical symptoms appear key to ensuring that symptoms are addressed in their bodily, existential, and social dimensions.

## Author Contributions

Idea development and study design: Malene Missel, Karin Piil, Linda Kahr Andersen, Mary Jarden. Methodology development and interview guide: Malene Missel, Karin Piil, Linda Kahr Andersen, Maria Camilla Mathiasen, Camilla Corvinius, Pernille Orloff Donsel. Data collection (interviews): Malene Missel, Linda Kahr Andersen, Maria Camilla Mathiasen, Camilla Corvinius, Pernille Orloff Donsel. Data analysis and interpretation: Malene Missel, Linda Kahr Andersen. Discussions and further interpretation of findings: All authors. Theoretical interpretations: All authors. Initial manuscript drafting: Malene Missel, Linda Kahr Andersen. Critical revision and multiple reviews: All authors. Final manuscript approval: All authors.

## Funding

The authors have nothing to report.

## Ethics Statement

This study was approved by the Danish Data Protection Agency (p‐2023‐15293) and conducted in accordance with guidelines from the Danish Ethics Research Committee and the Helsinki II Declaration [70]. Participants were provided with written information detailing the study's purpose and their right to withdraw at any time without consequences for their treatment. Prior to interviews, participants provided oral and written informed consent, with the assurance that their interview data would be handled confidentially. All data were anonymized using identification codes to protect participant privacy.

## Consent

The patients involved in the study were provided with comprehensive information both verbally and in writing. They were assured that their data would be handled with utmost confidentiality, and any information that could be traced back to them would be pseudo‐anonymized. Patients were explicitly informed of their right to withdraw from the study at any point without impacting their ongoing treatment. Written consent was obtained from all patients.

## Conflicts of Interest

The authors declare no conflicts of interest.

## Data Availability

The first author/corresponding author (M.M.) and the last author (L.K.A.) have full control of all primary raw data (interview transcripts) and allow the journal to review our data if requested. The data generated and/or analysed during the current study are available from the first author on reasonable request. All raw data are written in Danish. Data are stored in a locked file cabinet in a locked room at the Copenhagen University Hospital as requested by the Danish Data Protection Agency.

## References

[1] A. Norlyk , B. Martinsen , P. Dreyer , and A. Haahr , “Why Phenomenology Came Into Nursing: The Legitimacy and Usefulness of Phenomenology in Theory Building in the Discipline of Nursing,” International Journal of Qualitative Methods 22 (2023): 16094069231210433.

[2] O. P. Wiggins and M. A. Schwartz , “Richard Zaner's Phenomenology of the Clinical Encounter,” Theoretical Medicine and Bioethics 26, no. 1 (2005): 73–87. 15850044 10.1007/s11017-004-4805-3

[3] M. Kelly , C. Svrcek , N. King , A. Scherpbier , and T. Dornan , “Embodying Empathy: A Phenomenological Study of Physician Touch,” Medical Education 54, no. 5 (2020): 400–407. 31793673 10.1111/medu.14040

[4] J. Han , B. Frush , and J. R. Malone , “Loneliness in Medicine and Relational Ethics: A Phenomenology of the Physician‐Patient Relationship,” Clinical Ethics 19, no. 2 (2024): 171–181.

[5] M. Linden , U. Linden , D. Goretzko , and J. Gensichen , “Prevalence and Pattern of Acute and Chronic Multimorbidity Across All Body Systems and Age Groups in Primary Health Care,” Scientific Reports 12, no. 1 (2022): 272. 34997129 10.1038/s41598-021-04256-x PMC8742001

[6] G. Mahara , C. Tian , X. Xu , and W. Wang , “Revolutionising Health Care: Exploring the Latest Advances in Medical Sciences,” Journal of Global Health 13 (2023): 03042. 37539846 10.7189/jogh.13.03042 PMC10401902

[7] M. Missel , L. K. Andersen , C. Corvinius , et al., “Understanding Symptoms in the Lives of Adult Patients With Acute or Chronic Illness: A Phenomenological Study of Patient Experiences,” International Journal of Qualitative Studies on Health and Well‐Being 20, no. 1 (2025): 2534871, 10.1080/17482631.2025.2534871. 40736357 PMC12312141

[8] D. Baglow , K. Johnston , and M. Williams , “Existential Aspects of Breathlessness in Serious Disease,” Current Opinion in Supportive and Palliative Care 18, no. 4 (2024): 183–190. 39494535 10.1097/SPC.0000000000000736

[9] J. L. Larsen , E. O. C. Hall , S. Jacobsen , and R. Birkelund , “The Existential Experience of Everyday Life With Systemic Lupus Erythematosus,” Journal of Advanced Nursing 74, no. 5 (2018): 1170–1179. 29350776 10.1111/jan.13525

[10] E. C. Tarbi and S. H. Meghani , “A Concept Analysis of the Existential Experience of Adults With Advanced Cancer,” Nursing Outlook 67, no. 5 (2019): 540–557. 31040052 10.1016/j.outlook.2019.03.006 PMC6764914

[11] E. A. Hvidt , N. C. Hvidt , V. Graven , et al., “An Existential Support Program for People With Cancer: Development and Qualitative Evaluation,” European Journal of Oncology Nursing 46 (2020): 101768. 32446197 10.1016/j.ejon.2020.101768

[12] C. A. Laranjeira , P. P. Leao , and I. Leal , “The ‘Silenced’ Voices of Women Cancer Survivors: Bodily Experiences From an Existential Perspective,” Research and Theory for Nursing Practice 27, no. 3 (2013): 173–192. 24422332 10.1891/1541-6577.27.3.173

[13] M. Missel , H. Bergenholtz , M. Beck , P. O. Donsel , and C. Simonÿ , “Understanding Existential Anxiety and the Soothing Nature of Nostalgia in Life With Incurable Esophageal Cancer: A Phenomenological Hermeneutical Investigation of Patient Narratives,” Cancer Nursing 45, no. 1 (2021): E291–E298. 10.1097/NCC.0000000000000916 33443956

[14] M. Missel , J. H. Pedersen , C. Hendriksen , M. Tewes , and L. Adamsen , “Regaining Familiarity With Own Body After Treatment for Operable Lung Cancer—A Qualitative Longitudinal Exploration,” European Journal of Cancer Care 25, no. 6 (2016): 1076–1090. 26361265 10.1111/ecc.12383

[15] H. Carel , “Phenomenology as a Resource for Patients,” Journal of Medicine and Philosophy 37, no. 2 (2012): 96–113. 22474139 10.1093/jmp/jhs008

[16] I. J. Kidd and H. Carel , “Epistemic Injustice and Illness,” Journal of Applied Philosophy 34, no. 2 (2017): 172–190. 28303075 10.1111/japp.12172 PMC5324700

[17] H. H. Carel , “Phenomenology of Illness,” Phenomenological Reviews 3 (2017): 1–248.

[18] C. Lindström , E. Lindberg , A. H. Sandvik , and J. Karlsson , “Existential Reflections Expressed by Patients in Need of Hospital Care: A Qualitative Study,” Scandinavian Journal of Caring Sciences 39 (2025): e70047. 40551297 10.1111/scs.70047 PMC12185798

[19] J. Bradshaw , N. Siddiqui , D. Greenfield , and A. Sharma , “Kindness, Listening, and Connection: Patient and Clinician Key Requirements for Emotional Support in Chronic and Complex Care,” Journal of Patient Experience 9 (2022): 23743735221092627. 35434291 10.1177/23743735221092627 PMC9008851

[20] H. Pappot and G. A. Taarnhøj , “Expectations to Patient‐Reported Outcome (PRO) in Oncology–PRO for a Purpose, When and How?,” Acta Oncologica 59 (2020): 611–612. 32253960 10.1080/0284186X.2020.1749880

[21] M. G. Christiansen , H. Pappot , P. T. Jensen , M. R. Mirza , M. Jarden , and K. Piil , “A Multi‐Method Approach to Selecting PRO‐CTCAE Symptoms for Patient‐Reported Outcome in Women With Endometrial or Ovarian Cancer Undergoing Chemotherapy,” Journal of Patient‐Reported Outcomes 7, no. 1 (2023): 72. 37462855 10.1186/s41687-023-00611-w PMC10354345

[22] M. G. Christiansen , K. Piil , and M. Jarden , “The Symptom Experience and Self‐Management Strategies of Women Undergoing Cervical Cancer Treatment: A Qualitative Study,” Cancer Nursing 45, no. 1 (2021): 12–20. 10.1097/NCC.0000000000000843 32675630

[23] K. Piil , S. Laegaard Skovhus , A. Tolver , and M. Jarden , “Neuro‐Oncological Symptoms: A Longitudinal Quantitative Study of Family Function, Perceived Support, and Caregiver Burden,” Journal of Family Nursing 28, no. 1 (2022): 43–56. 34286624 10.1177/10748407211029986

[24] K. Piil , M. Juhler , J. Jakobsen , and M. Jarden , “Daily Life Experiences of Patients With a High‐Grade Glioma and Their Caregivers: A Longitudinal Exploration of Rehabilitation and Supportive Care Needs,” Journal of Neuroscience Nursing 47, no. 5 (2015): 271–284. 10.1097/JNN.0000000000000158 26348432

[25] L. K. Andersen , A. S. Jakobsson , K. L. Revsbech , and J. Vissing , “Causes of Symptom Dissatisfaction in Patients With Generalized Myasthenia Gravis,” Journal of Neurology 269, no. 6 (2022): 3086–3093. 34806129 10.1007/s00415-021-10902-1

[26] H. Carel , “Phenomenology and Its Application in Medicine,” Theoretical Medicine and Bioethics 32, no. 1 (2011): 33–46. 21103940 10.1007/s11017-010-9161-x

[27] H. Carel , “Pathology as a Phenomenological Tool,” Continental Philosophy Review 54, no. 2 (2021): 201–217. 34720686 10.1007/s11007-021-09538-9 PMC8550070

[28] M. Missel and R. Birkelund , “Ricoeur's Narrative Philosophy: A Source of Inspiration in Critical Hermeneutic Health Research,” Nursing Philosophy 21 (2019): e12254. 31087495 10.1111/nup.12254

[29] P. Ricoeur , Interpretation Theory: Discourse and the Surplus of Meaning. 8. pr. Fort Worth (Texas Christian University Press, 1976).

[30] P. Ricoeur , En Hermeneutisk Brobygger. Tekster Af Paul Ricoeur. [A Hermeneutic Bridge. Texts of Paul Ricoeur], ed. J. D. Rendtorff and, M. Hermansen (Forlaget Klim, 2002).

[31] D. F. Polit and C. T. Beck , Nursing Research: Generating and Assessing Evidence for Nursing Practice, 11th ed. (Wolters Kluwer, 2020).

[32] P. Ricoeur , “Eksistens og Hermeneutikk,” in Thorleif Dahls Kulturbibliotek (Aschehoug, 1999).

[33] P. Ricoeur , Time and Narrative, Vol. 1, ed. D. K. McLaughlin Pellauer (University of Chicago Press, 1984).

[34] The Danish National Center for Ethics , “Danish Research Ethics Committees,” 2024, https://researchethics.dk/.

[35] P. Ricoeur , “Sprog, Tale Og Skrift [Speech and Writing],” in Indlæg på filosofisk Grundtvig‐kongres, Københavns Universitet 9–10 September [Presentation at the Philosophical Grundtvig‐Congress, Copenhagen University 9–10 September], ed. P. Kemp (AROS, 1984).

[36] P. Ricoeur , Hermeneutics and the Human Sciences, 2nd ed., ed. J. B. Thompson (Cambridge University Press, 1998).

[37] P. Ricoeur , Filosofiens Kilder (Sources of Philosophy), ed. P. Kemp (Vintens Forlag, 1973).

[38] I. Scholl , J. M. Zill , M. Härter , and J. Dirmaier , “An Integrative Model of Patient‐Centeredness‐A Systematic Review and Concept Analysis,” PLoS One 9 (2014): e107828. 25229640 10.1371/journal.pone.0107828 PMC4168256

[39] V. Entwistle , A. Cribb , and I. Watt , “Shared Decision‐Making: Enhancing the Clinical Relevance,” Journal of the Royal Society of Medicine 105, no. 10 (2012): 416–421. 23104944 10.1258/jrsm.2012.120039 PMC3480854

[40] R. M. Epstein and R. L. Street , “The Values and Value of Patient‐Centered Care,” Annals of Family Medicine 9 (2011): 100–103. 21403134 10.1370/afm.1239 PMC3056855

[41] M. Missel , R. Petersen , E. Secher , et al., “Navigating Thymectomy With Myasthenia Gravis: A Qualitative Study on Patient Treatment Experiences,” Applied Nursing Research 83 (2025): 151961. 40473363 10.1016/j.apnr.2025.151961

[42] L. Ljungholm , A. Edin‐Liljegren , M. Ekstedt , and C. Klinga , “What Is Needed for Continuity of Care and How Can We Achieve It?—Perceptions Among Multiprofessionals on the Chronic Care Trajectory,” BMC Health Services Research 22, no. 1 (2022): 686. 35606787 10.1186/s12913-022-08023-0 PMC9125858

[43] F. Geese and K. U. Schmitt , “Interprofessional Collaboration in Complex Patient Care Transition: A Qualitative Multi‐Perspective Analysis,” Healthcare 11, no. 3 (2023): 359. 36766934 10.3390/healthcare11030359 PMC9914692

[44] G. J. Bøge , P. M. Louise , and J. H. S. Christensen Nana Zarthine , “Borgernes sundhedsvæsen,” 2016, www.regioner.dk.

[45] L. Thrysøe and R. Birkelund , “Sygepleje og klinisk beslutningstagning,” in Omsorg og patientinvolvering i klinisk beslutningstagning, ed. H. Steen (FADL's Forlag, 2018), http://findresearcher.sdu.dk/portal/da/publications/omsorg‐og‐patientinvolvering‐i‐klinisk‐beslutningstagning(cfb7bce0‐6dfc‐4cad‐b121‐f0d7baab2b25).html.

[46] L. Thrysoee , L. Birkelund , and R. Birkelund , “A Qualitative Study of Clinical Decision‐Making in a Multidisciplinary Outpatient Clinic for Patients With Atrial Fibrillation—From the Patient's Perspective,” European Journal of Person Centered Healthcare 6, no. 4 (2018): 610.

[47] T. A. Hutchinson , “Whole Person Care,” in Whole Person Care (Springer, 2011).

[48] A. Coulter , Engaging Patients in Healthcare (Open University Press, 2011).

[49] P. C. Skovlund , S. Ravn , L. Seibaek , H. V. Thaysen , K. Lomborg , and B. K. Nielsen , “The Development of PROmunication: A Training‐Tool for Clinicians Using Patient‐Reported Outcomes to Promote Patient‐Centred Communication in Clinical Cancer Settings,” Journal of Patient‐Reported Outcomes 4, no. 1 (2020): 10. 32048085 10.1186/s41687-020-0174-6 PMC7013008

[50] J. Greenhalgh , S. Dalkin , K. Gooding , et al., “Functionality and Feedback: A Realist Synthesis of the Collation, Interpretation and Utilisation of Patient‐Reported Outcome Measures Data to Improve Patient Care,” Health Services and Delivery Research 5, no. 2 (2017): 1–280. 28121094

[51] J. M. Woldring , R. O. B. Gans , W. Paans , and M. L. Luttik , “Physicians and Nurses View on Their Roles in Communication and Collaboration With Families: A Qualitative Study,” Scandinavian Journal of Caring Sciences 37, no. 4 (2023): 1109–1122. 37248644 10.1111/scs.13185

[52] A. Oster , E. Wiking , G. Nilsson , and C. Olsson , “Patients' Expectations of Primary Health Care From Both Patients' and Physicians' Perspectives: A Questionnaire Study With a Qualitative Approach,” BMC Primary Care 25, no. 1 (2024): 128. 38658808 10.1186/s12875-024-02389-2 PMC11040877

[53] A. Pichelstorfer and A. Mol , The Logic of Care: Health and the Problem of Patient Choice (Routledge, 2008).

[54] M. Galletta , M. F. Piazza , S. L. Meloni , et al., “Patient Involvement in Shared Decision‐Making: Do Patients Rate Physicians and Nurses Differently?,” International Journal of Environmental Research and Public Health 19, no. 21 (2022): 14229. 36361109 10.3390/ijerph192114229 PMC9656720

[55] M. Tringale , G. Stephen , A. M. Boylan , and C. Heneghan , “Integrating Patient Values and Preferences in Healthcare: A Systematic Review of Qualitative Evidence,” BMJ Open 12, no. 11 (2022): e067268. 10.1136/bmjopen-2022-067268 PMC9677014 36400731

[56] N. Clavel , J. Paquette , V. Dumez , et al., “Patient Engagement in Care: A Scoping Review of Recently Validated Tools Assessing Patients' and Healthcare Professionals' Preferences and Experience,” Health Expectations 24 (2021): 1924–1935. 34399008 10.1111/hex.13344 PMC8628592

[57] K. Toombs , Disability (Prometheus Books, 2008).

[58] K. Toombs , The Meaning of Illness (Springer, 1992).

[59] I. J. Kidd and H. Carel , “The Predicament of Patients,” Royal Institute of Philosophy Supplement 89 (2021): 65–84.

[60] D. Degerman and H. Carel , “Silence in Illness,” Royal Institute of Philosophy Supplement (2025), In press.

[61] S. K. Toombs , “The Meaning of Illness: A Phenomenological Approach to the Patient‐Physician Relationship,” Journal of Medicine and Philosophy 12, no. 3 (1987): 219–240. 3668399 10.1093/jmp/12.3.219

[62] M. O. Machado , N. Y. C. Kang , F. Tai , et al., “Measuring Fatigue: A Meta‐Review,” International Journal of Dermatology 60 (2021): 1053–1069. 33301180 10.1111/ijd.15341

[63] L. K. Andersen , M. Aadahl , and J. Vissing , “Fatigue, Physical Activity and Associated Factors in 779 Patients With Myasthenia Gravis,” Neuromuscular Disorders 31, no. 8 (2021): 716–725. 34303571 10.1016/j.nmd.2021.05.007

[64] I. K. Penner and F. Paul , “Fatigue as a Symptom or Comorbidity of Neurological Diseases,” Nature Reviews Neurology 13 (2017): 662–675. 29027539 10.1038/nrneurol.2017.117

[65] Y. M. J. Goërtz , A. M. J. Braamse , M. A. Spruit , et al., “Fatigue in Patients With Chronic Disease: Results From the Population‐Based Lifelines Cohort Study,” Scientific Reports 11, no. 1 (2021): 20977. 34697347 10.1038/s41598-021-00337-z PMC8546086

[66] J. W. Creswell , Qualitative Inquiry & Research Design. Choosing Among Five Approaches, 3rd ed. (SAGE Publications, 2013).

[67] J. M. Morse , “Critical Analysis of Strategies for Determining Rigor in Qualitative Inquiry,” Qualitative Health Research 25 (2015): 1212–1222. 26184336 10.1177/1049732315588501

[68] S. Gallagher and D. Zahavi , The Phenomenological Mind, 3rd ed. (Routledge, 2020).

[69] D. G. Spence , “Supervising for Robust Hermeneutic Phenomenology: Reflexive Engagement Within Horizons of Understanding,” Qualitative Health Research 27, no. 6 (2017): 836–842. 26984363 10.1177/1049732316637824

[70] World Medical Association Declaration of Helsinki , “World Medical Association Declaration of Helsinki: Ethical Principles for Medical Research Involving Human Subjects,” Journal of the American Medical Association 310, no. 20 (2013): 2191–2194. 24141714 10.1001/jama.2013.281053

